# Role of Surgery in the Management of Liver Metastases From Gastrointestinal Stromal Tumors

**DOI:** 10.3389/fonc.2022.903487

**Published:** 2022-07-01

**Authors:** Anwei Xue, Xiaodong Gao, Yifeng He, Ping Shu, Xiaowu Huang, Jianyi Sun, Jiangshen Lu, Yingyong Hou, Yong Fang, Kuntang Shen

**Affiliations:** ^1^ Department of General Surgery, Zhongshan Hospital, Fudan University, Shanghai, China; ^2^ Department of Liver Surgery, Zhongshan Hospital, Fudan University, Shanghai, China; ^3^ Department of Pathology, Zhongshan Hospital, Fudan University, Shanghai, China

**Keywords:** gastrointestinal stromal tumor, liver metastasis, surgery, imatinib, survival

## Abstract

**Background:**

The clinical benefit of hepatectomy in patients with liver metastases from gastrointestinal stromal tumors (GIST) has not been well defined in this era of tyrosine kinase inhibitor (TKI). Our study aims to demonstrate the survival advantage of adding hepatectomy in patients with GIST liver metastases.

**Methods:**

Information on patients with metastatic GIST treated or consulted between January 2006 and December 2018 was retrieved. Patients without extrahepatic metastases were included and classified into the surgical (S group) and non-surgical (NS group). Clinicopathological features were compared and their association with survival was assessed.

**Results:**

A total of 119 patients were included in this retrospective analysis, 62 in the S group and 59 in the NS group. Comparison of clinicopathological features showed that a markedly higher proportion of patients in the S group had ≤3 hepatic lesions (79.0% vs. 29.8%, *p*<0.001). After a median follow-up duration of 56 months, patients in the S group had significantly better progression-free survival (PFS) and marginally improved overall survival (OS) than those in the NS group (3y PFS:86.2% vs. 64.6%, *p*=0.002; 5y OS: 91.5% vs. 78.3%, *p*=0.083). After propensity score matching, multivariate analysis identified hepatectomy as the only significant prognostic factor for PFS while age, hepatectomy and max tumor diameter were significant predictor for OS.

**Conclusions:**

Addition of hepatectomy provided longer disease control in patients with metastatic GIST confined to the liver. Upfront hepatectomy followed by imatinib therapy is worthwhile trying in patients with single and easily removable lesions.

## Introduction

Gastrointestinal stromal tumors (GIST) constitute the most frequent mesenchymal malignancy of the gastrointestinal tract, with an estimated incidence of 10–20 per million population (1, 2). Surgery is the only curative modality for GIST, yet about 15–50% of newly-diagnosed patients present with metastatic disease, most frequently in the liver (3, 4). Even in patients with localized GIST completely removed, approximately half would experience recurrence in 18 to 24 months (5). Before the introduction of tyrosine kinase inhibitors (TKI), surgical resection had long been the only treatment expected to prolong the life of patients with metastatic GIST, but yielded a dismal 5-year overall survival (OS) rate of 27–34% (6, 7). Imatinib mesylate, the first-line TKI for treating GIST, has been highly effective in patients with advanced/metastatic disease and tripled the median survival duration to nearly 5 years (8). Controversy thus arose over the necessity of surgery and imatinib therapy has been proposed as a substitute considering its effectiveness and non-invasiveness. Despite a high response rate of up to 80%, complete response is rarely observed while the majority of patients progress on imatinib therapy (9). Unfortunately, the efficacy of subsequent lines of TKI in treating refractory GIST is limited, with a median progression-free survival (PFS) of 6.8 months for sunitinib and 4.8 months for regorafenib (10, 11).

Since disease progression on imatinib is inevitable, addition of surgical resection to reduce the risk of acquired resistance became an attractive investigatory approach. Though the therapeutic effect of hepatectomy is widely recognized in patients with liver metastases from colorectal carcinoma or neuroendocrine tumors (12, 13), there are limited data on the clinical benefit of hepatectomy in treating metastatic GIST in the era of imatinib. Most retrospective studies performed were underpowered due to their vague entry criteria and imbalanced patient characteristics. In the current study, we sought to evaluate the survival benefit of adding hepatectomy in patients receiving imatinib for liver metastases from GIST.

## Materials and Methods

### Patient Selection

The study protocol was approved by the institutional review board of Zhongshan Hospital, Fudan University and the requirement for informed consent was waived due to its retrospective nature. Patients who received surgical resection, imatinib medication or medical consultation for metastatic GIST in our institution from January 2006 through December 2018 were identified from the institutional medical records. The inclusion criteria were as follows (1): complete resection of primary tumors (2), biopsy-proven or radiological evidence of metastases confined to the liver (3), no history of local recurrence or distant metastasis, (4) receiving imatinib therapy for metastatic GIST, and (5) adequate liver, kidney and bone marrow function. We excluded patients with liver metastases emerging during imatinib therapy, age <18 years, prior malignancy and severe underlying disorders.

### Evaluation of Primary and Metastatic GIST

The electronic patient records were searched for information regarding patient characteristics (age and gender), primary tumors (location, interval from primary tumor resection to liver metastases), liver metastases (onset, number and location of metastatic tumors, max tumor diameter, response to preoperative imatinib, and date of hepatectomy). According to the time of occurrence, liver metastases were classified as synchronous (simultaneous with primary GIST or within 6 months after the diagnosis of primary tumors) or metachronous (beyond 6 months after the diagnosis of primary tumors).

### Surgical Management

For patients with resectable lesions, the choice whether to undergo hepatectomy was made by patients and surgeons jointly. Patients who had liver metastases primarily unresectable or needing extensive R0 organ resection but exhibited positive response to imatinib therapy were re-evaluated for surgery by dynamic radiological examinations. Major hepatectomy was defined as resection of three or more hepatic segments while every other resection was designated as minor hepatectomy. Surgical margins were classified as microscopically complete (R0), macroscopically complete with microscopic residual tumor cells (R1), or macroscopically incomplete (R2). Radiofrequency ablation was applied for highly-suspected or unfavorably located lesions and its curative effect was confirmed by contrast-enhanced computed tomography (CT) or magnetic resonance imaging (MRI). Postoperative complications were scored according to the Clavien-Dindo classification, with grade III and above considered major (14).

### Treatment With TKIs

Patients who had unresectable lesions or refused surgery were offered imatinib therapy after the diagnosis of liver metastases was made by liver biopsy or radiographic examination. Response to imatinib was assessed by contrast-enhanced CT or MRI at every 2 to 3 months and classified according to the Choi criteria (15). PFS was calculated as the length of time from the initiation of imatinib for liver metastases or date of hepatectomy to the date of documented recurrence or progression. OS was defined as the length of time from the date of imatinib administration or surgery for liver metastases to the date of last follow-up or tumor-related death, which ever occurred first. All the patients enrolled were mainly followed up on an outpatient basis or *via* telephone.

### Statistical Analyses

Continuous data are expressed as medians with ranges and compared using the Mann-Whitney *U* test. Categorical data were compared using Pearson’s chi-squared test or Fisher’s exact test, as appropriate. To minimize the impact of selection bias, potential pre-treatment parameters that may influence treatment decision were included in a 1:1 propensity score matching (PSM) with a caliper width of 0.1. Survival curves were plotted using the Kaplan–Meier method and intergroup differences were compared using the log-rank test. Univariate and multivariate analyses of the association between survival and potential prognostic factors were performed using the Cox proportional hazard regression models. All statistical analyses were performed using SPSS version 20.0 (SPSS Inc., Chicago, IL, USA). A two-tailed *p* value of less than 0.05 was considered statistically significant.

## Results

### Identification of Study Population

**Figure 1:**
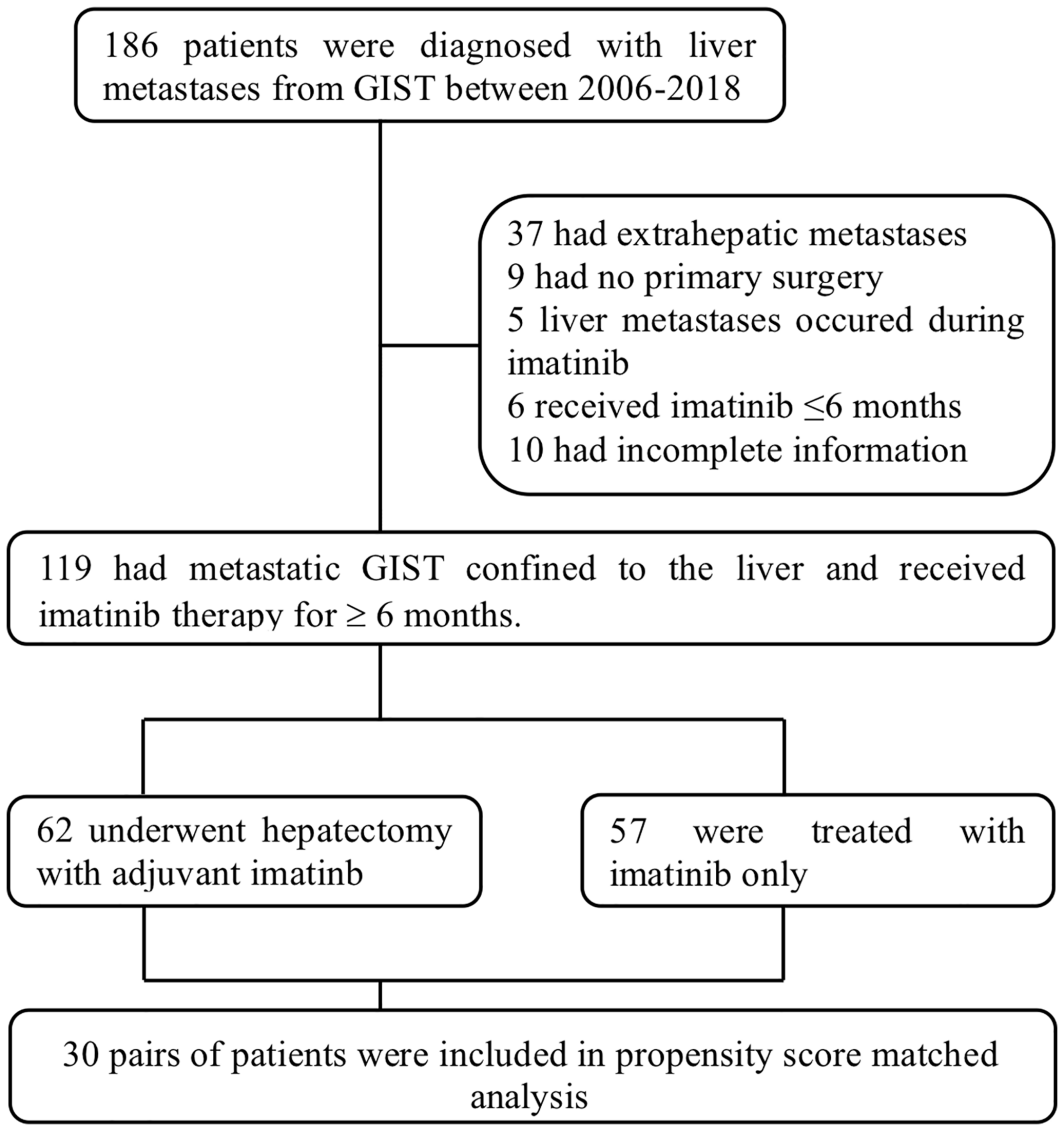
Flow chart of the patients included in this study.

Between January 2006 and December 2018, 186 patients were diagnosed with hepatic metastases from GIST in our institution and they were further reviewed for eligibility. Thirty-seven patients were then excluded for the existence of previous or simultaneous extrahepatic metastases, 9 excluded for not having undergone surgical resection of primary tumors, 5 excluded for refractory liver lesions emerging during imatinib therapy, 6 excluded for less than 6 months of imatinib administration and 10 excluded for incomplete follow-up or medical information (**Figure 1**). A total of 119 patients were ultimately included in this study, 62 in the surgical group (S group) and 57 in the non-surgical group (NS group).

### Patient Characteristics

Liver metastases were diagnosed in 71 (59.7%) males and 48 (40.3%) females at a median age of 56 (range, 26–82) years. Primary tumors were located in the small intestine in 67 (56.3%) patients, stomach in 46 (38.7%) patients and elsewhere in 6 (5.0%) patients. 44 (40.0%) patients presented with initially metastatic GISTs (synchronous) and the other 75 (60.0%) patients experienced liver metastasis more than 6 months after primary tumor resection (metachronous). The number of metastases were three or fewer in 66 (55.5%) patients and four or more in 53 (44.5%) patients, with a median diameter of the largest lesion measuring 4 (range 0.5–20.4) cm. Mutational analyses revealed mutations of *c-kit* exon 11 in 87 (77.7%) patients, *c-kit* exon 9 in 19 (17.0%) patients and *c-kit/PDGFRα* other exons in 3 (2.7%) patients. A comparison of baseline clinicopathological features between the surgical and non-surgical groups is shown in **Table 1**. A markedly higher proportion of patients in the surgical group had three or fewer metastatic nodules in the liver (79.0% vs. 29.8%, *p*<0.001).

**Table 1:** Baseline clinical characteristics of all patients.

Parameters	Surgery+Imatinib (n = 62)	Imatinib (n = 57)	*p*-value
Gender			
Male	34 (54.8%)	37 (64.9%)	0.263
Female	28 (45.2%)	20 (35.1%)	
Age (years)			
≤60	35 (56.5%)	34 (59.6%)	0.724
>60	27 (43.5%)	23 (40.3%)	
Primary sites			
Stomach	26 (41.9%)	20 (35.1%)	0.745
Small intestine	33 (53.2%)	34 (59.6%)	
Others	3 (4.9%)	3 (5.3%)	
Metastatic phase			
Synchronous	25 (40.3%)	19 (33.3%)	0.430
Metachronous	37 (59.7%)	38 (66.7%)	
No.of metastases			
≤3	49 (79.0%)	17 (29.8%)	<0.001
>3	13 (21.0%)	40 (70.2%)	
Largest diameter (cm)			
Median (range)	4.5 (0.6-20.4)	3.7(1-14.4)	0.235
≤4	28 (45.2%)	31 (54.4%)	0.315
>4	34 (54.8%)	26 (45.6%)	
Mutation analysis^a^			
*c-kit* exon 11	45 (76.3%)	42 (79.3%)	0.413
*c-kit* exon 9	12 (20.3%)	7 (13.2%)	
Other exons or none in *c-kit/PDGFRα*	2 (3.4%)	4 (7.5%)	

^a^Genetype results were unknown in 7 patients.

### Surgical Treatment and Outcomes

Of the 62 patients who received hepatectomy, resection of primary tumors was performed during the same procedure in 17 (27.4%) patients, ≤6 months before liver surgery in 7 (11.3%) patients and >6 months in 38 (61.3%) patients. Preoperative imatinib was administrated to 9 (14.5%) patients after the diagnosis of liver metastases, 7 (11.3%) of which exhibited partial response and 2 (3.2%) had stable disease. Thirty-eight (61.3%) patients underwent minor hepatectomy and 24 (38.7%) patients underwent major hepatectomy, while 9 (14.5%) patients received intraoperative radiofrequency ablation (RFA). R0 resection was achieved in 44 (71.0%) patients, R1 resection in 16 (25.8%) patients and R2 resection in 2 (3.2%) patients. Postoperative complications occurred in 8 (12.9%) patients, including abdominal infection in 3 (4.8%) patients, pleural effusion in 2 (3.2%) patients, anastomotic leakage in 1 (1.6%) patient, wound infection in 1 (1.6%) patient and encapsulated effusion in 1 (1.6%) patient. Invasive intervention was required in only one case while the rest were successfully managed by conservative treatment. No postoperative mortality was observed within 30 days after surgery and the median time from surgery to imatinib resumption was 31 days. Details of the surgical outcomes of patients are shown in **Table 2**.

**Table 2:** Surgical treatments and outcome.

Total number	62
Resection of primary GIST	
>6 months before liver surgery	38 (61.3%)
≤6 months before liver surgery	7 (11.3%)
During the same procedure	17 (27.4%)
Response to preoperative imatinib	
Partial response	7 (11.3%)
Stable disease	2 (3.2%)
Type of hepatectomy	
Minor (≤2 segments)	38 (61.3%)
Major (>2 segments)	24 (38.7%)
Locoregional intervention	
Intraoperative RFA	9 (14.5%)
Pre-/postoperative RFA/TACE	7 (11.3%)
Resection margin status	
R0	44 (71.0%)
R1	16 (25.8%)
R2	2 (3.2%)
Postoperative complications	
Anastomotic leakage	1 (1.6%)
Wound infection	1 (1.6%)
Encapsulated effusion	1 (1.6%)
Abdominal infection	3 (4.8%)
Pleural effusion	2 (3.2%)

RFA, radiofrequency ablation; TACE, transarterial chemoembolization.

### Survival Analysis

**Figure 2:**
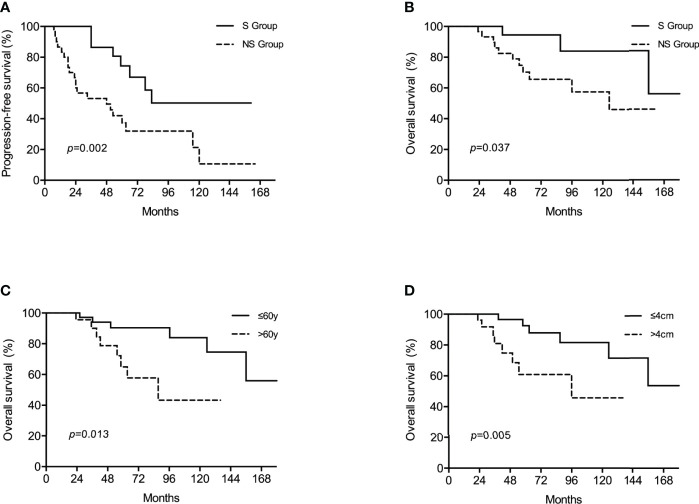
Kaplan–Meier survival analyses showed that in the propensity model, surgery was associated with improved progression-free survival (PFS) **(A)** while surgery **(B)**, age **(C)** and diameter of the largest nodule **(D)** were prognostic factors for overall survival (OS).

In the non-surgical group, partial response was discovered in 34 (59.6%) patients and stable disease in 22 (38.6%) patients. Liver metastases in 1 (1.8%) patient underwent complete cystic change and was considered complete response. After a median follow-up period of 56 (range, 24-189) months, 14 (22.6%) patients developed recurrence in the surgical group while 35 (61.4%) patients experienced disease progression in the non-surgical group, resulting in a 3-year PFS of 86.2% versus 64.6% (*p*=0.002). The 5-year OS rate were estimated to be 91.5% for the surgical group, marginally better than 78.3% for the non-surgical group (*p*=0.083). Following a 1:1 propensity score matching for age, metastatic phase, number of metastases and max tumor diameter, 30 pairs of patients with well-matched baseline features were selected (**Table 3**). Patients who underwent surgery in the propensity model still had significantly better PFS and OS when compared with those receiving imatinib only. The estimated 3-year PFS rate was 81.8% for the surgical group and 53.1% for the non-surgical group (*p*=0.001). The estimated 5-year OS rate in the surgical group was 94.4% and in the non-surgical group it was 70.3% (*p*=0.037). Survival graphs are shown in **Figure 2**. Hepatectomy was the only significant predictor for PFS in both the univariate and multivariate analyses. Among the risk factors associated with OS, multivariate analysis identified age, surgery and max tumor diameter as significant prognostic factors (**Table 4**).

**Table 3:** Baseline clinical characteristics of patients after propensity score matching.

Parameters	Surgery+Imatinib (n = 30)	Imatinib (n = 30)	*p*-value
Gender			
Male	15 (50.0%)	18 (60.0%)	0.436
Female	15 (50.0%)	12 (40.0%)	
Age (years)			
≤60	20 (66.7%)	17 (56.7%)	0.426
>60	10 (33.3%)	13 (43.3%)	
Primary sites			
Stomach	11 (36.7%)	12 (40.0%)	0.885
Small intestine	16 (53.3%)	16 (53.3%)	
Others	3 (10.0%)	2 (6.7%)	
Metastatic phase			
Synchronous	13 (43.3%)	9 (30.0%)	0.284
Metachronous	17 (56.7%)	21 (70.0%)	
No.of metastases			
≤3	17 (56.7%)	17 (56.7%)	1
>3	13 (43.3%)	13 (43.3%)	
Largest diameter (cm)			
≤4	20 (66.7%)	17 (56.7%)	0.426
>4	10 (33.3%)	13 (43.3%)	
Mutation analysis^a^			
*c-kit* exon 11	20 (71.4%)	22 (75.9%)	0.704
Non-*c-kit* exon 11	8 (28.6%)	7 (24.1%)	

^a^Genetype results were unknown in 3 patients.

**Table 4:** Prognostic factors for progression-free survival (PFS) and overall survival (OS) after propensity score matching.

Parameters	PFS			OS		
	*p*-value (univariate)	*p*-value (multivariate)	95% CI	*p*-value (univariate)	*p*-value (multivariate)	95% CI
Gender						
Male	0.342	–	1.445 (0.677-3.084)	0.280	–	1.799 (0.620-5.217)
Female						
Age(years)						
≤60	0.263	–	1.529 (0.726-3.219)	0.013	0.043	3.351 (1.038-10.818)
>60						
Primary sites						
Stomach	0.693	–	1.164 (0.548-2.470)	0.314	–	1.817 (0.568-5.811)
Others						
Metastatic phase						
Synchronous	0.693	–	1.167 (0.541-2.520)	0.748	–	0.840 (0.289-2.439)
Metachronous						
No.of metastases						
≤3	0.309	–	0.676 (0.317-1.438)	0.728	–	0.823 (0.273-2.475)
>3						
Largest diameter (cm)						
≤4	0.159	–	1.708 (0.811-3.595)	0.005	0.034	3.435 (1.101-10.716)
>4						
Mutation analysis						
*c-kit* exon 11	0.134	–	1.887 (0.822-4.330)	0.157	–	2.371 (0.718-7.827)
Non-*c-kit* exon 11						
Metastasectomy						
Yes	0.004	0.004	3.340 (1.476-7.559)	0.037	0.039	4.019 (1.076-15.010)
No						

## Discussion

Liver is the most frequent site of metastasis for GIST and liver metastases, once treated exclusively by surgery, pose a major threat to the survival of patients with GIST (5). The advent of imatinib has radically altered the management of GIST and greatly improved the survival of patients with GIST (16). Unlike the case in colorectal carcinoma and neuroendocrine tumors, where chemotherapy and targeted drugs act in synergy with surgery to improve survival, imatinib has more often superseded hepatectomy in patients with GIST liver metastases in view of its safety and effectiveness. Nonetheless, most patients on imatinib therapy eventually develop drug resistance and succumb to disease progression.

Although there is no direct evidence to support the role of surgery in delaying imatinib resistance, it is reasonable to deduce that the incidence of drug resistance is in proportion to the amount of tumor cells exposed and the duration of imatinib administration (17). Since continuous medication of imatinib is the cornerstone for disease control, surgical intervention to eliminate or reduce tumor burden seems a logical strategy. In addition, metastases in the liver are easier to identify and assess than those in the peritoneum, which makes them more appropriate for complete surgical resection. Hepatectomy was thus taken into consideration again to prolong the activity of imatinib. A Japanese prospective, multicenter trial was once conducted to clarify the efficacy and safety of surgery for liver oligometastasis but was prematurely terminated due to the amendment of the guideline and poor accrual (18). Several retrospective studies conducted in the last decade have indicated survival benefit from adding hepatectomy in the treatment of metastatic GIST, but were limited statistically by small sample size as well as their heterogeneity (19–26). To better demonstrate the clinical benefit of hepatectomy, we adopted strict inclusion criteria and collected a relatively homogenous cohort of patients with liver metastases from GIST.

At initial analysis of all patients, we found that hepatectomy plus imatinib was associated with improved PFS compared with imatinib alone (3y PFS, 86.2% vs. 64.6%, *p*=0.002). There was a tendency for better OS in the surgical group but this difference did not reach level of statistical significance (5y OS, 91.5% vs. 78.3%, *p*=0.083). Comparison of baseline clinicopathological characteristics between the two groups revealed that patients undergoing hepatectomy were more likely to have three or fewer metastatic nodules in the liver (79.0% v.s 29.8%, *p*<0.001). Along with age, metastatic phase and diameter of the largest nodule, factors that may affect the decision for surgery, a 1:1 propensity score matching was performed to minimize the impact of selection bias. In the propensity model, addition of surgery was still associated with better PFS and OS in both univariate and multivariate analyses. Previous studies reported a 3y PFS of 25.6%-77.5% for patients receiving surgery for liver metastases from GIST, which is inferior to our result (17, 19, 22, 25, 26). The difference in survival is probably attributed to the inclusion of patients with more advanced disease such as extrahepatic metastases and imatinib resistance in previous studies. All patients enrolled in the current study, however, were diagnosed with liver metastases for the first time and had no previous or simultaneous extra-hepatic lesions. Besides, all patients had primary tumors completely removed and received more than 6 months of imatinib for liver metastases.

According to the aforementioned assumption that metastectomy would delay acquired resistance to imatinib, surgical resection should be performed as completely as possible to minimize residual tumor cells. Bauer et al. provided evidence that patients with macroscopic complete metastectomy (R0/R1) had a longer median OS compared to those with incomplete metastectomy (R2) (8.7 versus 5.3 years) (23). A retrospective analysis performed by Seesing et al. showed that R0 resection was the only independent prognostic factor for PFS and OS in patients undergoing hepatectomy for GIST liver metastases (22). The impact of surgical margin status was not assessed in the current study due to our high rate of R0/R1 resection but we agree that a thorough preoperative examination is indispensable to avoid unnecessary debulking surgery. Another concern associated with metastectomy is that postoperative complications may counteract the survival benefit as a prolonged recovery would preclude the administration of imatinib. The rate of postoperative complications in our study turned out to be acceptable, with complications of any grade observed in 8 (12.9%) patients. There was no significant difference in postoperative complication between patients undergoing combined or sequential surgery (13.3% vs. 11.7%, *p*>0.05). Percutaneous peritoneal drainage was required in one case while the rest were successfully managed by conservative treatment. No postoperative mortality was observed within 30 days after surgery and the median time from surgery to imatinib resumption was 31 days.

Given that imatinib may shrink the tumor volume of GIST, its preoperative use has been proposed to minimize the extent of resection and the surgical morbidity (27, 28). Several retrospective analyses suggested that response to preoperative imatinib, partially responsive or stable, were associated with prolonged survival in patients with metastatic GIST (29, 30). One possible explanation is that patients with liver metastases from GIST are more likely to achieve R0 resection after downsizing imatinib therapy (31). In the real world, however, it is not uncommon for patients with responsive disease to refuse surgery and return with signs of disease progression. Frequent surveillance helps to detect early signs of progression, for which surgical resection is still possible and beneficial, but general progression unfortunately contraindicates surgical intervention (30, 32). Therefore, for patients with primarily unresectable tumors or needing extensive R0 organ resection, the focus should be on identifying the optimal timing of surgery and duration of preoperative imatinib, weighing survival benefit against risk of acquired resistance.

The management of initially resectable liver metastases is, on the other hand, different from their “less easily removable” counterparts. There are two strategies for this subset of patients according to the sequence of surgery. Preoperative imatinib followed by surgical resection is appropriate for patients with resectable “high volume” tumors. Upfront liver resection followed by adjuvant imatinib therapy is the other option to be considered, particularly for single and easily removable lesions. This approach is helpful to relieve patients of the need to undergo frequent preoperative radiological reassessment and may also reduce risk of acquired drug resistance by minimizing tumor cells exposed to imatinib exposure. In our series, 53 patients (85.5%) in the surgical group underwent hepatectomy prior to imatinib administration and R0/R1 resection were achieved in 98.1% of cases. The 3y PFS rate was 86.6% for patients with upfront hepatectomy and 83.3% for those responsive to preoperative imatinib. A prospective, real-world, observational study is ongoing in our institution to demonstrate the safety and effectiveness of upfront hepatectomy followed by imatinib in patients with initially resectable liver metastases from GIST (ChiCTR2000035773).

Despite being the largest study with a relatively strict inclusion criteria, our study has several limitations. First, patients with fewer number of metastases were prone to undergo surgery, a selection bias that cannot be avoided in this retrospective study. After propensity score matching, patient characteristics were balanced but the number of cases was relatively small. Second, as a high-volume center with experienced surgeons specialized in liver surgery, patients with liver metastases were more likely to receive surgical resection in our institution, partly accounting for the low proportion of patients receiving preoperative imatinib. Third, the information on treatment modalities after progression or recurrence was unknown in some cases, which is important for survival analysis.

## Conclusions

Hepatectomy combined with imatinib seems to offer better survival than imatinib alone in patients with metastases confined to the liver. Upfront hepatectomy should be considered an option for patients with initially resectable liver metastases, especially single and easily removable lesions.

## Data Availability Statement

The original contributions presented in the study are included in the article/supplementary materials. Further inquiries can be directed to the corresponding authors.

## Ethics Statement

The studies involving human participants were reviewed and approved by Institutional review board of Zhongshan Hospital, Fudan University. Written informed consent for participation was not required for this study in accordance with the national legislation and the institutional requirements.

## Author Contributions

KS and YF conceptualized and designed this retrospective study. AX and XG performed most of the statistical analyses and wrote the draft manuscript. JS, JL, and PS assisted with the data collection. YFH, XH, and YYH made substantive intellectual contributions to the paper. All authors read and approved the final manuscript.

## Funding

We thank the support of National Natural Science Foundation of China (No. 81773080) and Clinical Research Plan of Shanghai Hospital Development Center (No. SHDC 2020CR4038) for this study.

## Conflict of Interest

The authors declare that the research was conducted in the absence of any commercial or financial relationships that could be construed as a potential conflict of interest.

## Publisher’s Note

All claims expressed in this article are solely those of the authors and do not necessarily represent those of their affiliated organizations, or those of the publisher, the editors and the reviewers. Any product that may be evaluated in this article, or claim that may be made by its manufacturer, is not guaranteed or endorsed by the publisher.

## References

[1] Chan KH Chan CW Chow WH Kwan WK Kong CK Mak KF. Gastrointestinal Stromal Tumors in a Cohort of Chinese Patients in Hong Kong. World J Gastroenterol (2006) 12(14):2223–8. 10.3748/wjg.v12.i14.2223 PMC4087650 16610025

[2] Tryggvason G Gislason HG Magnusson MK Jonasson JG. Gastrointestinal Stromal Tumors in Iceland, 1990-2003: The Icelandic GIST Study, a Population-Based Incidence and Pathologic Risk Stratification Study. Int J cancer (2005) 117(2):289–93. 10.1002/ijc.21167 15900576

[3] Miettinen M Lasota J. Gastrointestinal Stromal Tumors. Gastroenterol Clinics North America (2013) 42(2):399–415. 10.1016/j.gtc.2013.01.001 PMC3644178 23639648

[4] Nilsson B Bumming P Meis-Kindblom JM Oden A Dortok A Gustavsson B. Gastrointestinal Stromal Tumors: The Incidence, Prevalence, Clinical Course, and Prognostication in the Preimatinib Mesylate Era–a Population-Based Study in Western Sweden. Cancer (2005) 103(4):821–9. 10.1002/cncr.20862 15648083

[5] Dematteo RP Lewis JJ Leung D Mudan SS Woodruff JM Brennan MF. Two Hundred Gastrointestinal Stromal Tumors: Recurrence Patterns and Prognostic Factors for Survival. Ann surgery (2000) 231(1):51. 10.1097/00000658-200001000-00008 PMC1420965 10636102

[6] DeMatteo RP Shah A Fong Y Jarnagin WR Blumgart LH Brennan MF. Results of Hepatic Resection for Sarcoma Metastatic to Liver. Ann Surgery (2001) 234(4):540–7. doi: 10.1097/00000658-200110000-00013 PMC1422077 11573047

[7] Nunobe S Sano T Shimada K Sakamoto Y Kosuge T. Surgery Including Liver Resection for Metastatic Gastrointestinal Stromal Tumors or Gastrointestinal Leiomyosarcomas. Japanese J Clin Oncol (2005) 35(6):338–41. doi: 10.1093/jjco/hyi091 15928191

[8] Keung EZ Raut CP Rutkowski P. The Landmark Series: Systemic Therapy for Resectable Gastrointestinal Stromal Tumors. Ann Surg Oncol (2020) 27(10):3659–71. doi: 10.1245/s10434-020-08869-w PMC7471171 32734368

[9] Antonescu CR Besmer P Guo T Arkun K Hom G Koryotowski B. Acquired Resistance to Imatinib in Gastrointestinal Stromal Tumor Occurs Through Secondary Gene Mutation. Clin Cancer Res an Off J Am Assoc Cancer Res (2005) 11(11):4182–90. doi: 10.1158/1078-0432.CCR-04-2245 15930355

[10] Demetri GD van Oosterom AT Garrett CR Blackstein ME Shah MH Verweij J. Efficacy and Safety of Sunitinib in Patients With Advanced Gastrointestinal Stromal Tumour After Failure of Imatinib: A Randomised Controlled Trial. Lancet (London England) (2006) 368(9544):1329–38. doi: 10.1016/S0140-6736(06)69446-4 17046465

[11] Demetri GD Reichardt P Kang YK Blay JY Rutkowski P Gelderblom H. Efficacy and Safety of Regorafenib for Advanced Gastrointestinal Stromal Tumours After Failure of Imatinib and Sunitinib (GRID): An International, Multicentre, Randomised, Placebo-Controlled, Phase 3 Trial. Lancet (London England) (2013) 381(9863):295–302. 10.1016/S0140-6736(12)61857-1 PMC3819942 23177515

[12] Rees M Tekkis PP Welsh FK O'Rourke T John TG. Evaluation of Long-Term Survival After Hepatic Resection for Metastatic Colorectal Cancer: A Multifactorial Model of 929 Patients. Ann Surgery (2008) 247(1):125–35. 10.1097/SLA.0b013e31815aa2c2 18156932

[13] Mayo SC de Jong MC Pulitano C Clary BM Reddy SK Gamblin TC. Surgical Management of Hepatic Neuroendocrine Tumor Metastasis: Results From an International Multi-Institutional Analysis. Ann Surg Oncol (2010) 17(12):3129–36. 10.1245/s10434-010-1154-5 20585879

[14] Dindo D Demartines N Clavien PA. Classification of Surgical Complications: A New Proposal With Evaluation in a Cohort of 6336 Patients and Results of a Survey. Ann Surgery (2004) 240(2):205–13. 10.1097/01.sla.0000133083.54934.ae PMC1360123 15273542

[15] Choi H Charnsangavej C Faria SC Macapinlac HA Burgess MA Patel SR. Correlation of Computed Tomography and Positron Emission Tomography in Patients With Metastatic Gastrointestinal Stromal Tumor Treated at a Single Institution With Imatinib Mesylate: Proposal of New Computed Tomography Response Criteria. J Clin Oncol Off J Am Soc Clin Oncol (2007) 25(13):1753–9. 10.1200/JCO.2006.07.3049 17470865

[16] Vassos N Agaimy A Hohenberger W Croner RS. Management of Liver Metastases of Gastrointestinal Stromal Tumors (GIST). Ann Hepatol (2015) 14(4):531–9. doi: 10.1016/S1665-2681(19)31175-5 26019040

[17] Turley RS Peng PD Reddy SK Barbas AS Geller DA Marsh JW. Hepatic Resection for Metastatic Gastrointestinal Stromal Tumors in the Tyrosine Kinase Inhibitor Era. Cancer (2012) 118(14):3571–8. doi: 10.1002/cncr.26650 PMC3290751 22086856

[18] Kanda T Masuzawa T Hirai T Ikawa O Takagane A Hata Y. Surgery and imatinib therapy for liver oligometastasis of GIST: A study of Japanese Study Group on GIST. Japanese J of Clin Oncol (2017) 47(4):369–72. doi: 10.1093/jjco/hyw203 28073945

[19] Xiao B Peng J Tang J Zhang R Li C Lin J. Liver Surgery Prolongs the Survival of Patients With Gastrointestinal Stromal Tumor Liver Metastasis: A Retrospective Study From a Single Center. Cancer Manage Res (2018) 10:6121–7. doi: 10.2147/CMAR.S187061 PMC6257109 30538560

[20] Shi YN Li Y Wang LP Wang ZH Liang XB Liang H. Gastrointestinal Stromal Tumor (GIST) With Liver Metastases: An 18-Year Experience From the GIST Cooperation Group in North China. Med (Baltimore) (2017) 96(46):e8240. doi: 10.1097/MD.0000000000008240 PMC5704785 29145240

[21] Sato S Tsujinaka T Masuzawa T Yamamoto K Takahashi T Yamashita Y. Role of Metastasectomy for Recurrent/Metastatic Gastrointestinal Stromal Tumors Based on an Analysis of the Kinki GIST Registry. Surg Today (2017) 47(1):58–64. doi: 10.1007/s00595-016-1351-3 27194124

[22] Seesing MFJ Tielen R van Hillegersberg R van Coevorden F de Jong KP Nagtegaal ID. Resection of Liver Metastases in Patients With Gastrointestinal Stromal Tumors in the Imatinib Era: A Nationwide Retrospective Study. Eur J Surg Oncol (EJSO) (2016) 42(9):1407–13. doi: 10.1016/j.ejso.2016.02.257 27038995

[23] Bauer S Rutkowski P Hohenberger P Miceli R Fumagalli E Siedlecki JA. Long-Term Follow-Up of Patients With GIST Undergoing Metastasectomy in the Era of Imatinib – Analysis of Prognostic Factors (EORTC-STBSG Collaborative Study). Eur J Surg Oncol (2014) 40(4):412–9. doi: 10.1016/j.ejso.2013.12.020 24491288

[24] Sato S Tsujinaka T Yamamoto K Takahashi T Kishi K Imamura H. Primary Surgery as a Frontline Treatment for Synchronous Metastatic Gastrointestinal Stromal Tumors: An Analysis of the Kinki GIST Registry. Surg Today (2016) 46(9):1068–75. doi: 10.1007/s00595-015-1282-4 26611538

[25] Brudvik KW Patel SH Roland CL Conrad C Torres KE Hunt KK. Survival After Resection of Gastrointestinal Stromal Tumor and Sarcoma Liver Metastases in 146 Patients. J Gastrointestinal Surg Off J Soc Surg Alimentary Tract (2015) 19(8):1476–83. doi: 10.1007/s11605-015-2845-9 PMC4506212 26001368

[26] Cheung TT Chok KS Chan AC Yau TC Chan SC Poon RT. Analysis of Long-Term Survival After Hepatectomy for Isolated Liver Metastasis of Gastrointestinal Stromal Tumour. ANZ J Surg (2014) 84(11):827–31. 10.1111/ans.12249 23782558

[27] Raut CP Posner M Desai J Morgan JA George S Zahrieh D. Surgical Management of Advanced Gastrointestinal Stromal Tumors After Treatment With Targeted Systemic Therapy Using Kinase Inhibitors. J Clin Oncol Off J Am Soc Clin Oncol (2006) 24(15):2325–31. 10.1200/JCO.2005.05.3439 16710031

[28] Park SJ Ryu MH Ryoo BY Park YS Sohn BS Kim HJ. The Role of Surgical Resection Following Imatinib Treatment in Patients With Recurrent or Metastatic Gastrointestinal Stromal Tumors: Results of Propensity Score Analyses. Ann Surg Oncol (2014) 21(13):4211–7. 10.1245/s10434-014-3866-4 24980089

[29] Zaydfudim V Okuno SH Que FG Nagorney DM Donohue JH. Role of Operative Therapy in Treatment of Metastatic Gastrointestinal Stromal Tumors. J Surg Res (2012) 177(2):248–54. 10.1016/j.jss.2012.07.005 22831567

[30] Fairweather M Balachandran VP Li GZ Bertagnolli MM Antonescu C Tap W. Cytoreductive Surgery for Metastatic Gastrointestinal Stromal Tumors Treated With Tyrosine Kinase Inhibitors: A 2-Institutional Analysis. Ann Surgery (2018) 268(2):296–302. 10.1097/SLA.0000000000002281 PMC6203295 28448384

[31] DeMatteo RP Maki RG Singer S Gonen M Brennan MF Antonescu CR. Results of Tyrosine Kinase Inhibitor Therapy Followed by Surgical Resection for Metastatic Gastrointestinal Stromal Tumor. Ann Surgery (2007) 245(3):347–52. doi: 10.1097/01.sla.0000236630.93587.59 PMC1877004 17435539

[32] Gao X Xue A Fang Y Shu P Ling J Qin J. Role of Surgery in Patients With Focally Progressive Gastrointestinal Stromal Tumors Resistant to Imatinib. Sci Rep (2016) 6:22840. doi: 10.1038/srep22840 26946961 PMC4780000

